# Gallnut tannic acid alleviates gut damage induced by *Salmonella pullorum* in broilers by enhancing barrier function and modulating microbiota

**DOI:** 10.3389/fvets.2024.1382288

**Published:** 2024-05-28

**Authors:** Junjie Zou, Hongliang Luan, Pengyuan Xi, Junshu Xue, Jiahao Fan, Xinyi Zhong, Xun Zhou, Xu Song, Xinghong Zhao, Yuanfeng Zou, Lixia Li, Renyong Jia, Yuping Fu, Zhongxiu Liu, Zhongqiong Yin

**Affiliations:** ^1^Natural Medicine Research Center, College of Veterinary Medicine, Sichuan Agricultural University, Chengdu, China; ^2^Qilu Animal Health Products Co., Ltd., Jinan, China; ^3^Key Laboratory of Animal Disease and Human Health of Sichuan Province, Sichuan Agricultural University, Chengdu, China; ^4^Chengdu QianKun Veterinary Pharmaceutical Co., Ltd, Chengdu, China

**Keywords:** *Salmonella pullorum*, pullorum disease, tannic acid, gut barrier, intestinal flora

## Abstract

Pullorum disease (PD) is a bacterial infection caused by *Salmonella pullorum* (*S. pullorum*) that affects poultry. It is highly infectious and often fatal. Antibiotics are currently the mainstay of prophylactic and therapeutic treatments for PD, but their use can lead to the development of resistance in pathogenic bacteria and disruption of the host's intestinal flora. We added neomycin sulfate and different doses of tannic acid (TA) to the drinking water of chicks at 3 days of age and infected them with PD by intraperitoneal injection of *S. pullorum* at 9 days of age. We analyzed intestinal histopathological changes and the expression of immune-related genes and proteins by using the plate smear method, histological staining, real-time fluorescence quantitative PCR, ELISA kits, and 16S rRNA Analysis of intestinal flora. The results demonstrate that *S. pullorum* induces alterations in the immune status and impairs the functionality of the liver and intestinal barrier. We found that tannic acid significantly ameliorated *S. pullorum*-induced liver and intestinal damage, protected the intestinal physical and chemical barriers, restored the intestinal immune barrier function, and regulated the intestinal flora. Our results showed that TA has good anti-diarrhoeal, growth-promoting, immune-regulating, intestinal barrier-protecting and intestinal flora-balancing effects, and the best effect was achieved at an additive dose of 0.2%.

## 1 Introduction

Pullorum disease (PD) is a septicaemia that affects chickens, turkeys, and other poultry, caused by *Salmonella pullorum* infection. Clinical manifestations in chicks include anorexia, diarrhea, debilitation, reduced egg production, and increased mortality rate (1, 2). PD outbreaks can cause significant financial losses for the poultry farming industry in developing nations (3). Research has shown that *S. pullorum* invasion initially causes severe damage to chick intestinal function and microbiota and subsequently spreads to host tissues and organs, weaken immunity, causing septicaemia and compromising the immune responses (4–6). Tetracycline and kanamycin are commonly used drugs in the clinical treatment and prevention of PD. Nevertheless, prolonged use of these antibiotics can lead to the development of resistance in *S. pullorum* and disrupt the host's intestinal flora, resulting in serious residual problems (7–9). Therefore, it is essential to find a safe and effective alternative to antibiotics.

Tannins are naturally occurring polyphenolic compounds primarily known for their haemostatic, anti-infective, and anti-diarrhoeal properties (10, 11). Tannic acid (TA), the most basic form of hydrolyzed tannins, plays a crucial role in traditional Chinese medicines like gallnut. Recent research has revealed that tannic acid can significantly improve animal performance, regulate immunological function, and enhance resistance against disease (12–14). In addition, TA has been approved by regulatory authorities, such as the US Food and Drug Administration (FDA) (11) and the European Union (15), as a food additive and flavoring agent due to its potential antimicrobial properties. The results presented above demonstrate TA's effective antimicrobial activity and biosafety. Furthermore, TA has been shown to effectively reduce damage to the intestinal mucosa caused by oxidative stress and inflammatory responses (16, 17), improve gut microbial composition, and enhance intestinal barrier function (18–20). Therefore, TA may have a preventive effect against PD and could alleviate the intestinal damage caused by *S. pullorum*.

Brus et al. (21) demonstrated that concentrations of TA solutions ranging from 0.05 to 0.1% can promote the growth of small intestinal epithelial cells in chickens. However, no studies have investigated the use of tannic acid as a supplement in poultry drinking water for the treatment of PD. Moreover, the exact influence of TA on the function of the intestinal mucosal barrier and the composition of the intestinal flora remains incompletely comprehended. Thus, this study seeks to examine the consequences of tannic acid on the gut's morphological structure and barrier function in broilers infected with *S. pullorum*, and to investigate potential mechanisms associated with microbiota involvement.

## 2 Materials and methods

### 2.1 Materials

TA, extracted from gallnut, was purchased from Wufeng Chicheng Biotech Co., Ltd (content ≥ 95%, 20220309), quality requirements outlined in the FDA's “Food Chemical Specification” 5th edition and the U.S. Food Chemical Codex standards. Furthermore, it complied with the U.S. food FCC-IV standards.

### 2.2 Bacterial strains and growth conditions

The *S. pullorum* strain (CVCC1792) utilized in this study was acquired from the National Center for Veterinary Culture Collection (Beijing, China). The strain was activated in TSA medium and incubated at 37°C for 24 h. Following this, individual colonies were cultured in TSB medium and incubated at 37°C with a shaking speed of 180 rpm for 24 h. The cultured bacteria were then centrifuged (5,000 g/min, 5 min), washed three times with phosphate-buffered solution (PBS), and resuspended to achieve a final concentration of 1 × 10^9^ CFU/ml.

### 2.3 Animal care and *S. pullorum* challenge

A total of 240 1-day-old B380 broilers were purchased from Chengdu Tianxinli Poultry Co (Chengdu, China). The groups included: (1) the negative control group (NC), which did not receive TA treatment or *S. pullorum* infection; (2) the *S. pullorum* challenged group (SP), which received *S. pullorum* infection and 0% gallnut TA; (3) the positive control group (PC), which received *S. pullorum* infection and 0.04% Neomycin sulfate soluble powder in drinking water; (4) the TA low dose group (TAL), which received *S. pullorum* infection and 0.1% gallnut TA in drinking water; (5) the TA middle dose group (TAM), which received *S. pullorum* infection and 0.2% gallnut TA in drinking water; (6) the TA high dose group (TAH), which received *S. pullorum* infection and 0.3% gallnut TA in drinking water. In order to prevent cross-infection, various groups were segregated and maintained at a distance from one another's enclosures. At 3 days old, the administration was started, and lasted for a duration of 12 days.

At the age of 9 days, all groups, except for the NC group, were intraperitoneal injected with 0.5 ml of *S. pullorum* (1 × 10^9^ CFU/ml). The NC group received an equivalent volume of PBS. The diets and drinking water were kept consistent throughout the experiment. Clinical signs were monitored daily, and changes in weight and mortality rates were recorded for each group of chicks.

### 2.4 Sample collection

The mortality rate of chicks following the challenge was recorded to determine the survival rate. At the age of 10 and 14 days, 20 chicks were chosen at random from each group for weighing. Additionally, six chicks were randomly selected to have blood samples collected from their jugular vein, which were subsequently centrifuged to obtain serum. Following the collection of blood, the chicks were administered anesthesia andeuthanized. Subsequently, the organ lesions were dissected and examined. Immune organs were weighed, and the organ-to-body weight ratio was calculated. Liver samples were collected for bacterial load determination, with a portion being immersed in 4% paraformaldehyde. Two sections each of mid-jejunum and mid-ileum (1 cm each) were collected; one section was fixed in 4% paraformaldehyde, while the other was frozen at −80°C. Cecal contents were collected and stored in liquid nitrogen for subsequent 16S rRNA sequencing.

### 2.5 Bacterial burden of the liver

On days 1 and 5 post-infection, a small amount of liver was weighed and homogenized in 1 ml of PBS. Serial dilutions of the homogenate were then spread on XLD agar plates to determine the quantity of *S. pullorum*.

### 2.6 Histopathological examination of the liver and small intestine

The liver and 1 cm of the jejunum and ileum were collected, rinsed with PBS at 4°C, and fixed in 4% paraformaldehyde for 24 h at room temperature. Liver, jejunum, and ileum samples were then embedded in paraffin, sectioned at a thickness of 5 μm, and stained with hematoxylin-eosin (HE) for morphological analysis. Goblet cells in the jejunum were then counted using periodic acid-Schiff stain (PAS).

### 2.7 Determination of serum IL-4, IFN-γ, IgG, LPS, DAO, and jejunal mucosal sIgA

The concentrations of interleukin-4 (IL-4), interferon-γ (IFN-γ), immunoglobulin G (IgG), lipopolysaccharide (LPS), and Diamine oxidase (DAO) in serum, as well as sIgA in the jejunum, were determined using ELISA kits following the manufacturer's instructions (MlBio, Shanghai, China). The total protein concentration was assessed using the BCA assay kit (Beyotime, Shanghai, China).

### 2.8 Quantitative real-time PCR

Jejunum and tissue samples were collected on days 10 and 14. The samples were snap-frozen in liquid nitrogen and then transferred to −80°C for storage. Total RNA was extracted using TRIzol reagent (Biomed, Beijing, China) following the manufacturer's instructions. RNA (5 μg/μl) was reverse transcribed into cDNA using the M-MLV 4 First-Strand cDNA Synthesis Kit (Biomed, Beijing, China) and further applied to qRT-PCR in a step One plus System (Biosystems, Foster City, CA, USA) with double-stranded DNA. qRT-PCR was performed using Hieff UNICONR Universal Blue qPCR SYBR Green Master Mix (Yeasen Biotechnology Co., Ltd, Shanghai, China). The predenaturation procedure was 95°C for 30 s, followed by 40 cycles of 95°C for 10 s, and annealing/extension at the optimal annealing temperature for 30 s. All procedures were performed according to the manufacturer's instructions. The 2^−ΔΔCt^ method was used to analyze the relative levels of gene expression. Gene expression levels were normalized to the internal standard, β-actin. The primer sequences (Tsingke Biotechnology Co., Ltd, China) used for gene expression analysis in this study are listed in Table 1.

**Table 1:** Sequences of the oligonucleotide primers used for quantitative real-time PCR^a^.

**Gene name**	**GenBank accession No**.	**Primer (5^′^ → 3^′^)**
*Claudin 1*	NM_001013611.2	F: GCCAAGATCACCATC GTCTC
		R: CACCAGCGGGTTGTA GAAAT
*Occluding*	NM_205128.1	F: CTGCTGTCTGTGGG TTCCT
		R: CCAGTAGATGTTGG CTTTGC
*ZO-1*	NM_001301025.3	F: CTTCAGGTGTTTCTCTT CCTCCTC
		R: CTGTGGTTTCATGG CTGGATC
*MUC-2*	XM_040701656.2	F: CAAAAGCACCTAGCAC AACGA
		R: CTTAACAACTTCAC GGCACT
*IL-4*	NM_001007079.2	F: CATCTGCCTCCT ACCAC
		R: TTCTGATCTCGCATT ACGTT
*IL-10*	NM_001004414.4	F: ATGCTGCGCTTCT ACACA
		R: CCATGCTCTGCTG ATGACT
*IL-18*	NM_204608.3	F: TCTGGCAGTGGAATG TACTTCG
		R: CCATTTTCCCATGCTCT TTCTC
*IFN-γ*	NM_205149.2	F: CAAGTCAAAGCC GCACA
		R: TTTCACCTTCTTCAC GCCAT
*β-actin*	NM_205518.2	F: GTGACCTGACGGACT ACCTC
		R: TCTCCTGCTCGAAA TCCAGT

^a^Primers were designed and synthesized by Tsingke Biotechnology Co., Ltd. (China).

ZO-1, zonula occludens-1; MUC2, mucin 2; IL-4, interleukin-4; IL-10, interleukin-10; IL-18, interleukin-18; IFN-γ, *interferon*−γ.

### 2.9 16S rRNA gene sequencing of the cecum microbiome

The fresh digesta isolated from the cecum was quickly frozen in liquid nitrogen and then rapidly sent to Novogene Technology Co. (Beijing, China), Ltd. for intestinal microbiota analysis under dry ice conditions. The DNA was extracted using the CTAB/SDS (cetyltrimethylammonium bromide/sodium dodecyl sulfate) bromide method, and the concentration was detected by agarose gel electrophoresis, and then diluted to 1 ng/μl with sterile water. The V3–V4 region of 16S rRNA was amplified by PCR using specific primers: 341F(CCTAYGGGRBGCASCAG) and 806R(GGACTACNNGGGTATCTAAT). The process was followed by mixing and purification of PCR products, and library construction using the TruSeq^®^ DNA PCR-Free Sample Preparation Kit. The constructed libraries were quantified using Qubit and Q-PCR, and then sequenced on the NovaSeq 6000 platform.

### 2.10 Statistical analysis

Data represent the mean ± standard deviation (SD) or mean ± standard error of the mean (SEM). One-way ANOVA with LSD *post-hoc* test was performed by using SPSS 27.0 software. Significance was determined at *P* < 0.05.

## 3 Results

### 3.1 Survival rate and weight performance

**Figure 1:**
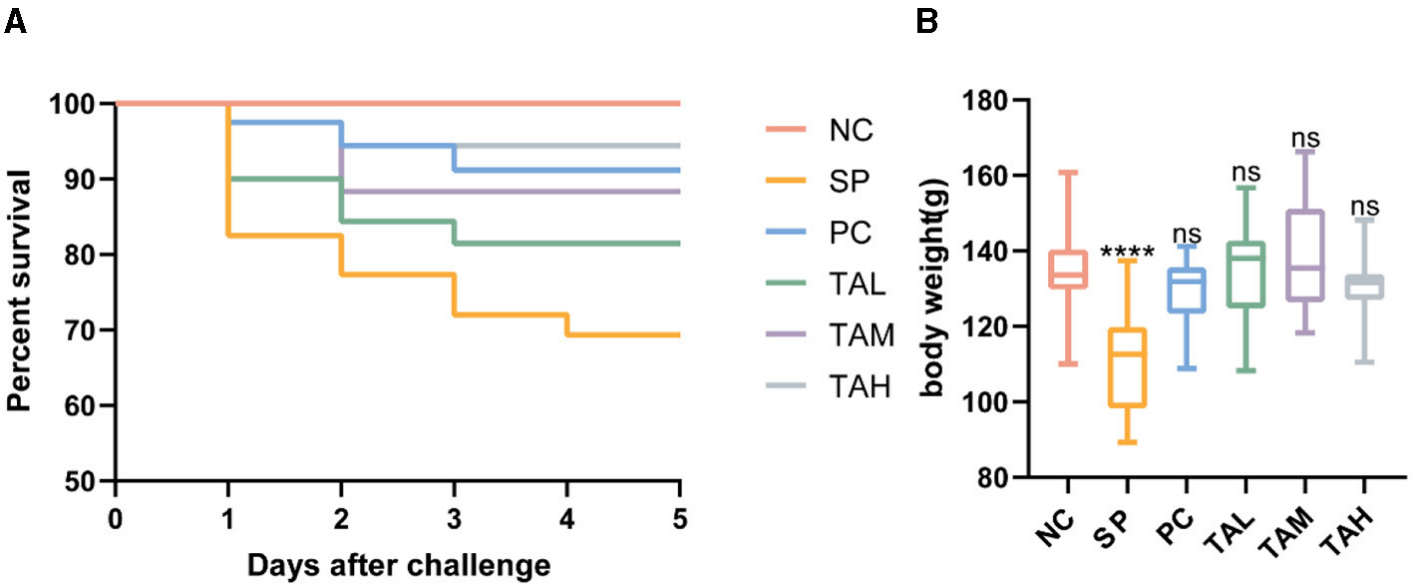
Effects of tannic acid supplementation on survival and body weight of broiler chickens. **(A)** Survival of broiler chickens (*n* = 40) supplemented with tannic acid. **(B)** Body weight of broiler chickens (*n* = 20) supplemented with tannic acid. NC, negative control. SP, *Salmonella pullorum* challenge; PC, positive control; TAL, low-dose tannic acid; TAM, medium-dose tannic acid; TAH, high-dose tannic acid. Data are mean ± SD. Results are mean ± SD. ****P < 0.0001 (one-way ANOVA); ns, not significant (compared with NC group).

The survival rate and body weight of broiler chickens infected with *S. pullorum* improved with the addition of tannic acid to their drinking water, as shown in Figure 1A. The SP group had a survival rate of 70%, while the PC, TAL, TAM, and TAH groups had survival rates of 92.5%, 82.5%, 90%, and 95%, respectively. On day 5 post-infection, the body weight of chicks in the SP group decreased by 18% to 110.71 g ± 13.18, compared to the NC group (135.00 g ± 11.25, *P* < 0.0001). However, the chicks' body weight was normalized by treatment with TA, and the most significant increase was observed in the TAM group (Figure 1B).

### 3.2 Organ index, liver bacterial load, and liver histomorphology

**Figure 2:**
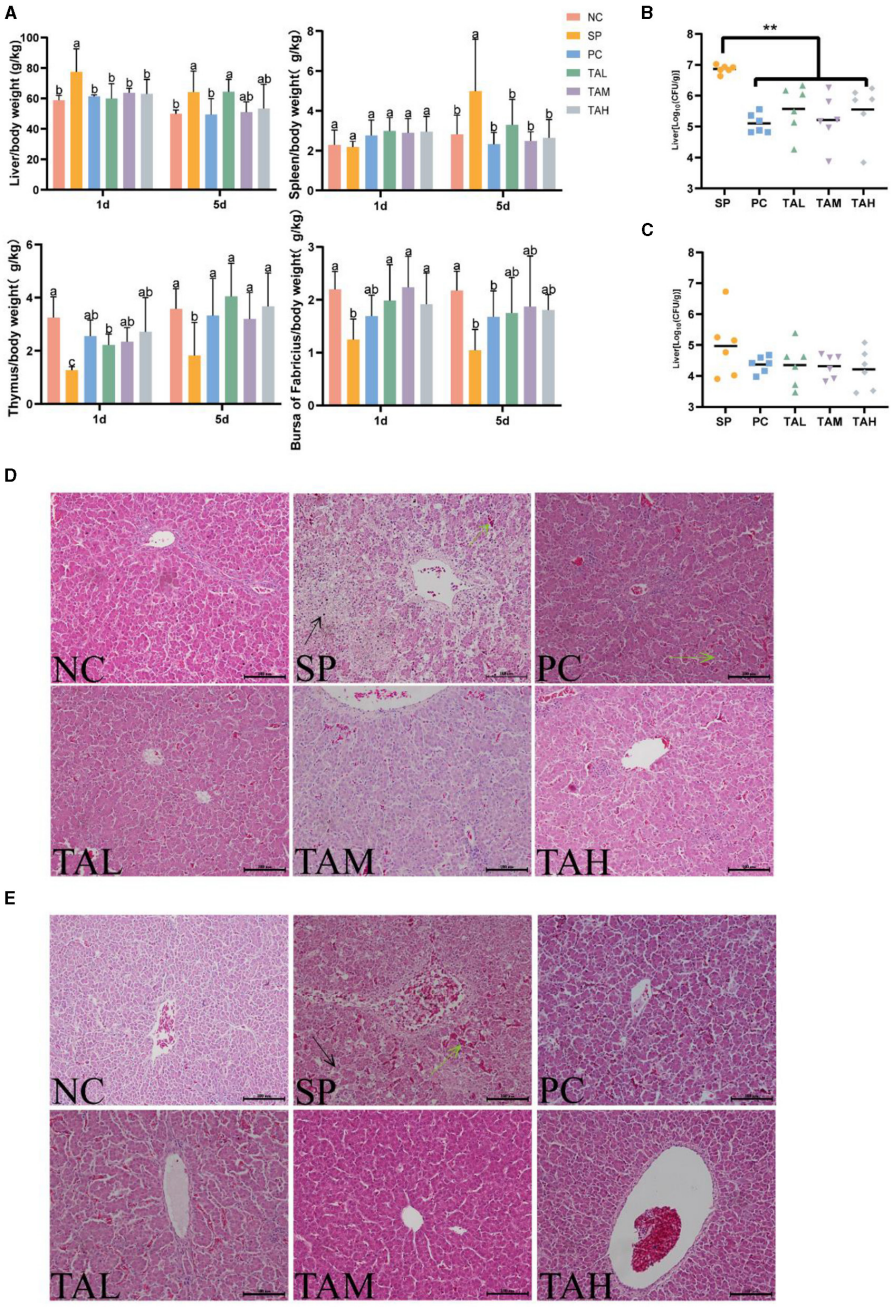
Organ-specific gravity, liver bacterial load, and pathological changes in broilers. Liver/body weight ratio and immune organ/body weight ratio on days 1 and 5 post-infection **(A)** (*n* = 6). Bacterial loads (log10 CFU per gram of *Salmonella pullorum*) in the liver on day 1 **(B)** and day 5 **(C)** post infection (*n* = 6). Pathology of the liver of chicks in different treatment groups on day 1 **(D)** and day 5 **(E)** post infection (scale bar = 100 μm). Data are expressed as mean ± SD. Results are presented as mean § SEM. Different letters indicate significant differences between groups (*P* < 0.05). **P < 0.01 (One-way ANOVA). → : areas of liver necrosis; → : congested areas of liver.

Figure 2 shows the organ index, liver bacterial load, and liver pathology findings. Chickens infected with *S. pullorum* had a significantly higher liver/body weight ratio on the first and fifth day of infection. In addition, there was a significant increase in the spleen/body weight ratio on the fifth day of infection. In contrast, the thymus/body weight ratios and bursa/body weight ratios were significantly reduced (*P* < 0.05; Figure 2A). On the first day of infection, supplementation of tannic acid resulted in a decrease in the bacterial load in the liver compared to the SP group, and returned the ratio of organ/body weight to normal levels (Figures 2A–C). The group treated with SP showed significant liver sinusoidal dilatation and extensive necrosis on the first day of the experiment. In contrast, the group treated with TA exhibited only mild hepatic inflammation and congestion. On day 5, the TAM and TAH groups showed almost normal liver morphology, except for the localized inflammation observed in the TAL group (Figures 2D, E). Nevertheless, the SP group still showed persistent liver damage and congestion.

### 3.3 Serum immunological index

**Figure 3:**
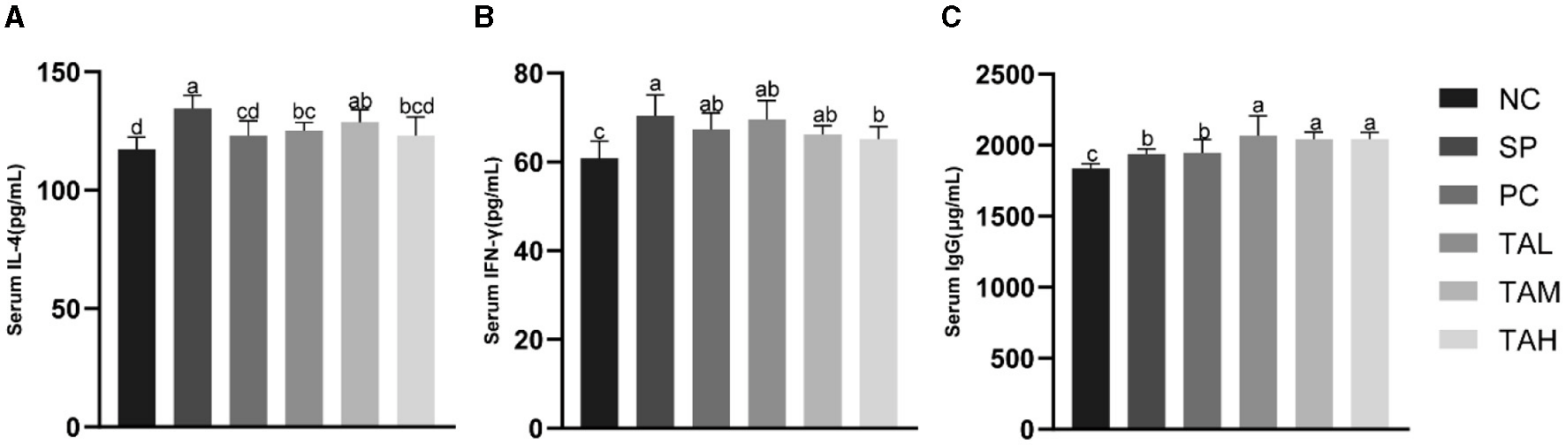
Levels of serum immunity indexes in broiler chickens at the 5th day of infection. **(A)** Serum levels of IL-4. **(B)** Serum levels of IFN-γ; **(C)** Serum levels of IgG. All data are expressed as the mean ± SD. The results are presented as the mean ± SD. Different letters indicate significant differences between the groups (*P* < 0.05).

Figure 3A shows the levels of serum cytokines IFN-γ, IL-4 and immunoglobulin IgG. The results indicate that *S. pullorum* infection significantly increased serum levels of IL-4, IFN-γ, and IgG on day 5 compared to the NC group (Figures 3A–C). Treatment with antibiotics and tannic acid reduced serum levels of IL-4 and IFN-γ in infected chicks, with the lowest levels observed in the TAH group (*P* < 0.05). Serum IgG levels remained high in all groups with TA compared to the NC group (*P* < 0.05) and were significantly higher than in the SP group (*P* < 0.05).

### 3.4 Intestinal histomorphology

**Figure 4:**
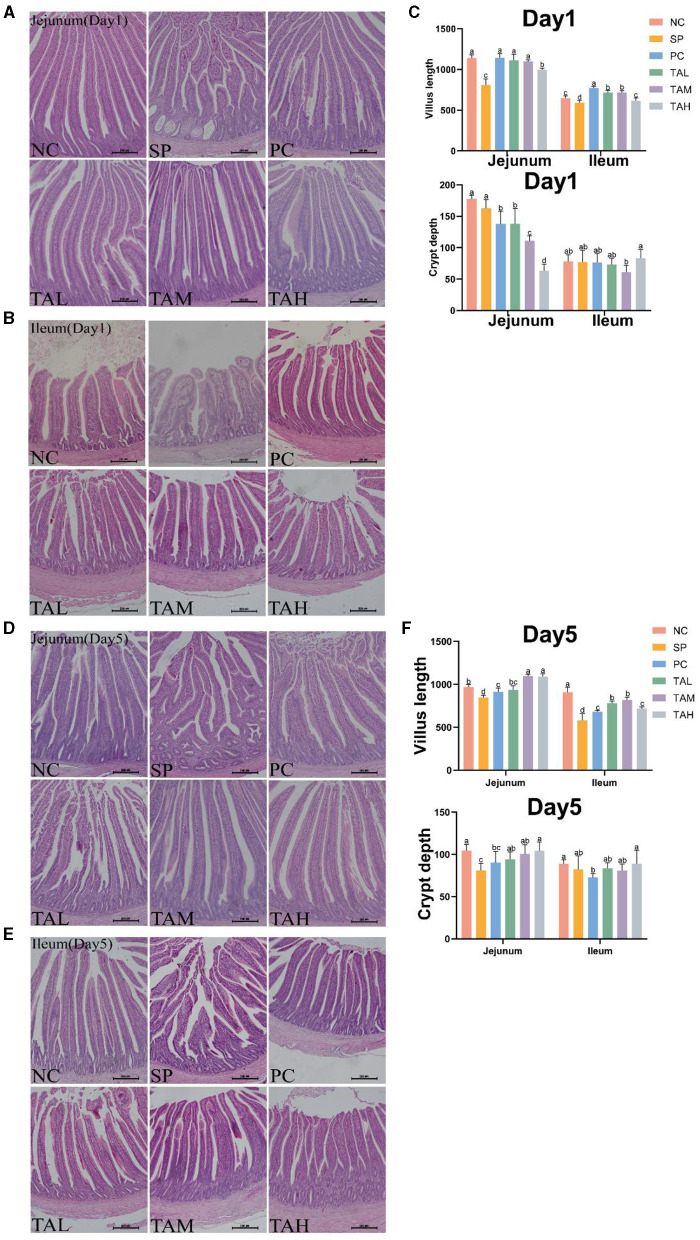
Pathologic sections of the jejunum and ileum of broiler chickens. **(A, B)** Representative images of H.E staining shows pathology of the jejunum and ileum in different treatment groups on day 1 post infection. **(C)** Statistics of villus height and crypt depth contents on day 1 post infection. **(D, E)** Representative images of H.E staining shows pathology of the jejunum and ileum in different treatment groups on day 5 post infection (scale bar =100 μ m). **(F)** Statistics of villus height and crypt depth contents on day 5 post infection. All data are expressed as the mean ± SD. The results are presented as the mean § SEM. Different letters indicate significant differences between the groups (*P* < 0.05).

Histopathological analysis showed that infection with *S. pullorum* resulted in significant damage to the jejunal and ileal villi in the SP group. The presence of broken villi, thickened crypts, and a decrease in the number of villi in the SP group of chicks on days 1 and 5, when compared to the NC group (Figures 4A, B, D, E). The chicks in the TAL, TAM, and TAH groups had a higher presence of intact intestinal structures and villi when compared to the SP group. The results of further analysis indicate that the heights of both jejunal and ileal villi were significantly lower in the SP group than in the NC group on days 1 and 5 (*P* < 0.05). Supplementation with TA effectively restored the height of these villi to normal levels (Figures 4C, F).

### 3.5 Jejunal goblet cell count

**Figure 5:**
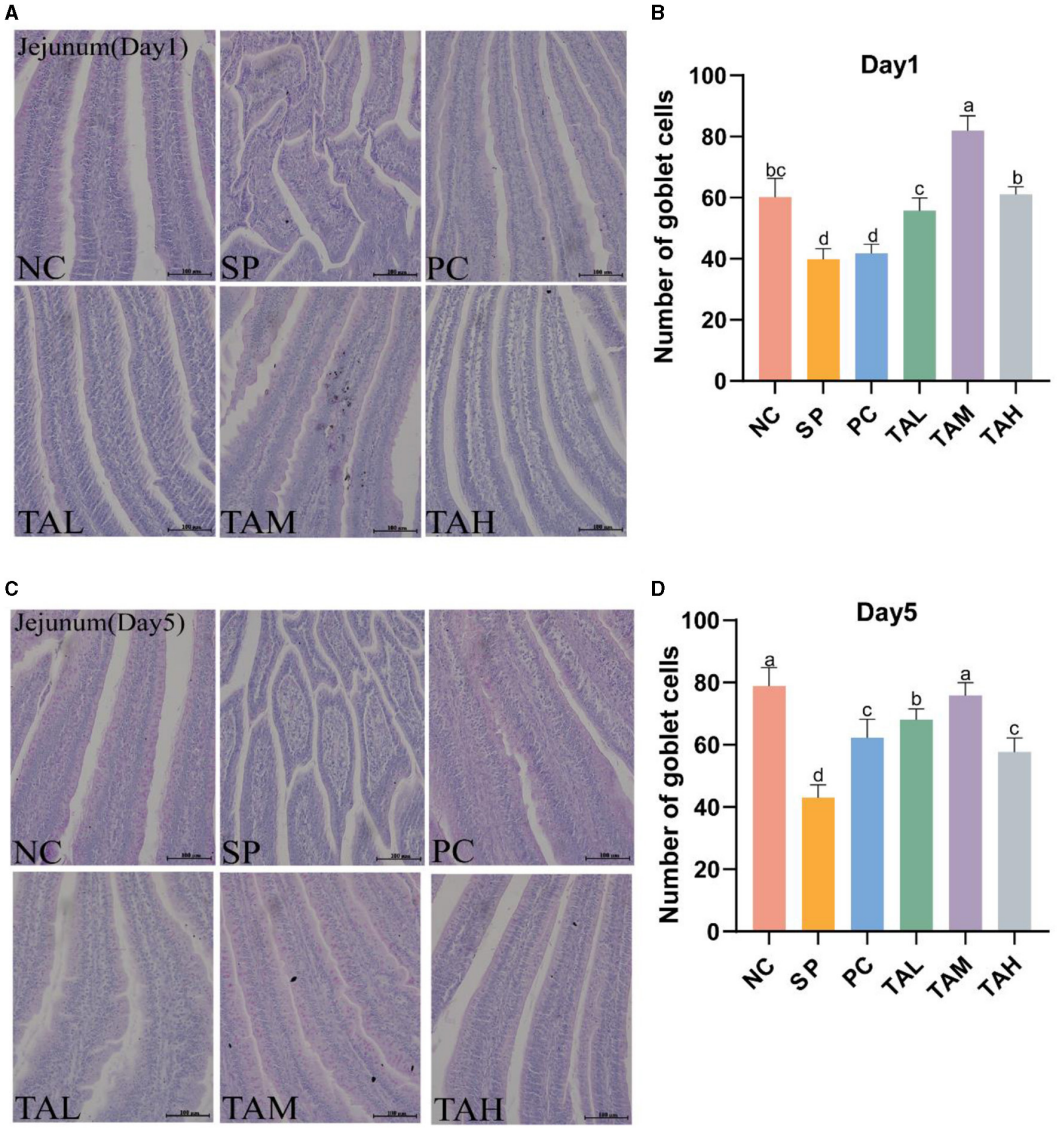
PAS staining of the jejunum. **(A, C)** PAS staining of the jejunum on days 1 and 5 post-infection (scale bar =100 μ m). **(B, D)** Statistics of goblet cells numbers of the jejunum in different groups on day 1 and day 5 post infection. All data are expressed as the mean ± SD. The results are presented as the mean ± SD. Different letters indicate significant differences between the groups (*P* < 0.05).

The PAS staining revealed that the goblet cells of the chick jejunum were stained red (Figures 5A, B). The SP group had fewer jejunal goblet cells than the NC group on both days 1 and 5 after *S. pullorum* infection (*P* < 0.05). On day 1, all TA groups had normal goblet cell numbers (*P* < 0.05). On day 5, all TA groups had more goblet cells than the SP group (*P* < 0.05), and there was no significant difference between the TAM group and the NC group (Figures 5C, D).

### 3.6 Expression of mRNA for intestinal barrier function genes and determination of serum LPS and DAO concentrations

**Figure 6:**
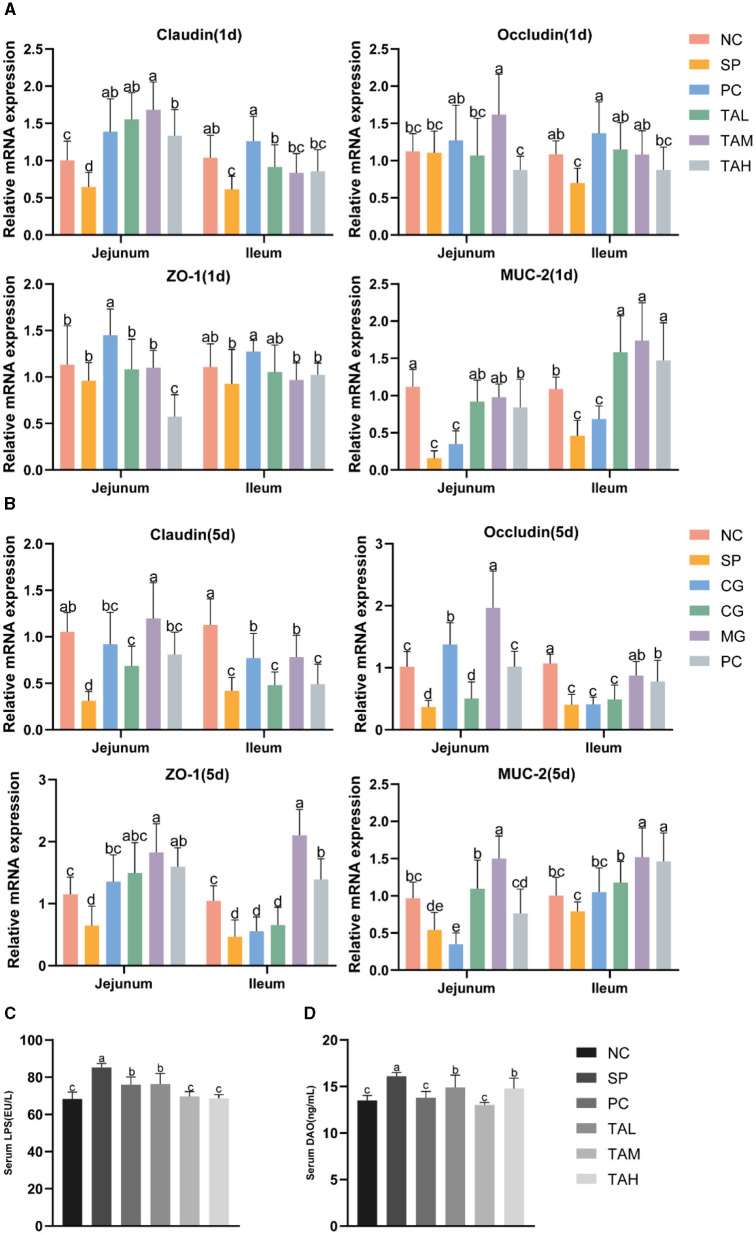
Relative expression levels from qRT-PCR and serum ELISA. **(A)** Expression of mRNA for Claudin-1, Occluding, ZO-1 and MUC-2 in the jejunum and ileum of chicks on day 1 post-infection (*n* = 4). **(B)** Expression of mRNA for Claudin-1, Occludin, ZO-1 and MUC-2 in the jejunum and ileum of chicks on day 1 post-infection (*n* = 4). **(C)** LPS levels in serum on the day 5 of infection. **(D)** DAO levels in serum on the day 5 of infection. All data are expressed as the mean ± SD. The results are presented as the mean § SEM. Different letters indicate significant differences between the groups (*P* < 0.05).

**Figure 7:**
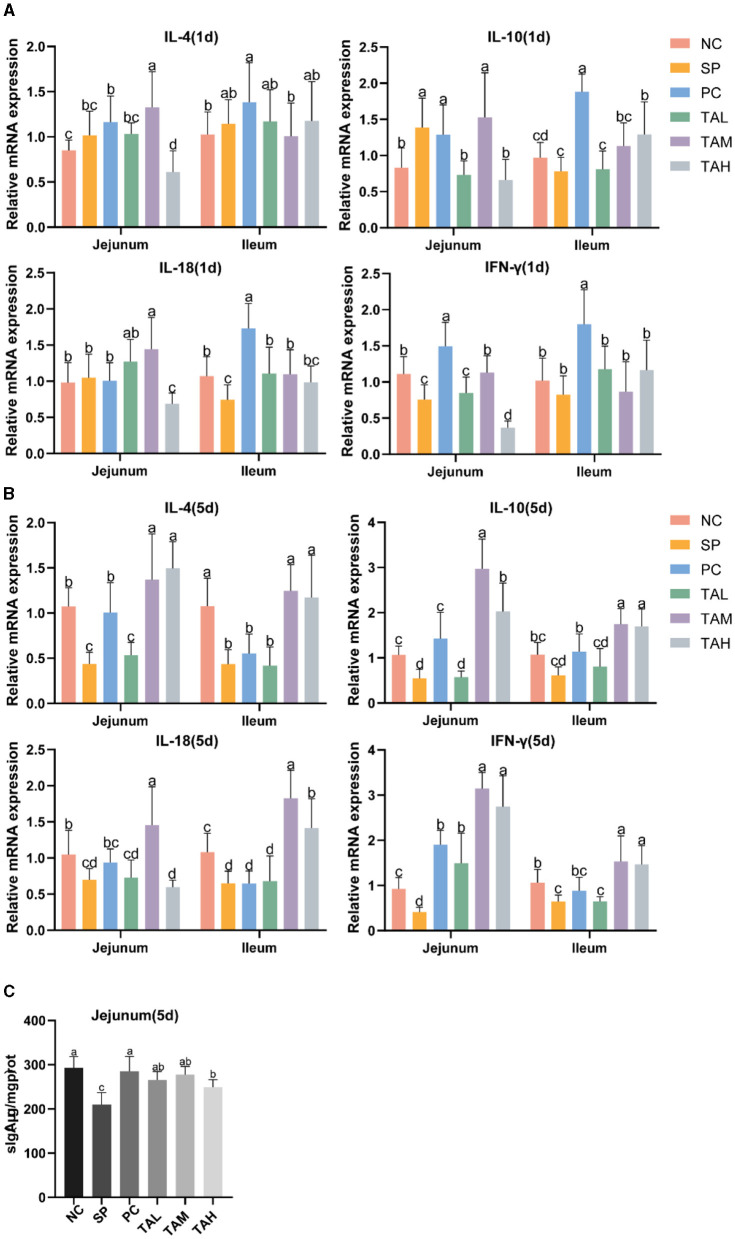
Relative expression levels from qRT-PCR and jejunum ELISA. **(A)** Expression of mRNA for IL-4, IL-10, IL-18 and IFN-γ in the jejunum and ileum of chicks on day 1 post-infection (*n* = 4). **(B)** Expression of mRNA for IL-4, IL-10, IL-18 and IFN-γ in the jejunum and ileum of chicks on day 5 post-infection (*n* = 4). **(C)** Levels of sIgA in jejunum on day 5 of infection (*n* = 5). All data are expressed as the mean ± SD. The results are presented as the mean ± SD. Different letters indicate significant differences between the groups (*P* < 0.05).

Figure 6 illustrates the impact of TA on intestinal tight junction proteins, mucin and permeability in broilers challenged with *S. pullorum*. On the day 1 of infection, the mRNA transcript levels of tight junction proteins (Claudin-1 and Occludin) and mucin (MUC-2) were significantly reduced in the jejunum and ileum of chicks in the SP group compared to the NC group. The expression of both tight junction proteins and mucins was markedly diminished on the fifth day of infection (*P* < 0.05). On the first and fifth days of infection, TA supplementation increased the mRNA transcript levels of tight junction proteins and mucins compared to the SP group. On the fifth day, the TAM group showed significantly higher levels of jejunal Occludin, ZO-1, and MUC2, as well as ileal ZO-1, compared to the NC group (*P* < 0.05). The TAL group showed lower levels of Claudin-1 and Occludin compared to the NC group. Figures 6A, B show that the PC and TAH groups had lower levels of Claudin-1 and higher levels of ileal ZO-1 and MUC2 compared to the NC group. Alterations in serum lipopolysaccharide (LPS) and diamine oxidase (DAO) levels were observed on the fifth day after infection. The SP group exhibited considerably elevated levels of LPS and DAO in comparison to the NC group (*P* < 0.05). TA can effectively reduce the blood levels of LPS and DAO in chicks, with the most significant effect observed in the TAM group. In the TAM group, serum levels of LPS and DAO returned to normal (Figures 6C, D). During the initial infection stage, IL-10 mRNA transcript levels significantly increased in the jejunum, while IFN-γ markedly decreased in both the jejunum and ileum of chicks in the SP group. The mRNA transcript levels of the cytokines IL-4, IL-18 and IFN-γ were found to be significantly higher in the TAM group than in the SP group (Figure 7A). On the fifth day of infection, the SP group showed significantly lower levels of IL-4, IL-10, IL-18, and IFN-γ in the jejunal and ileal regions compared to the NC group (*P* < 0.05). The cytokine levels in the PC, TAM, and TAH groups increased to varying degrees, except for the TAL group. The group subjected to TAM demonstrated the most significant promotion impact, as illustrated in Figure 7B. Secretory IgA (sIgA) is crucial for establishing and maintaining the immune barrier. On the fifth day after infection, we observed a 28.29% decrease in the level of sIgA in the jejunal tissue of the SP group compared to the NC group (*P* < 0.05). After the addition of tannic acid, the levels of jejunal sIgA in broilers were significantly higher than in the SP group. Only the TAH group showed a significant decrease compared to the NC group (Figure 7C).

### 3.7 Microbiological analysis of the cecum

**Figure 8:**
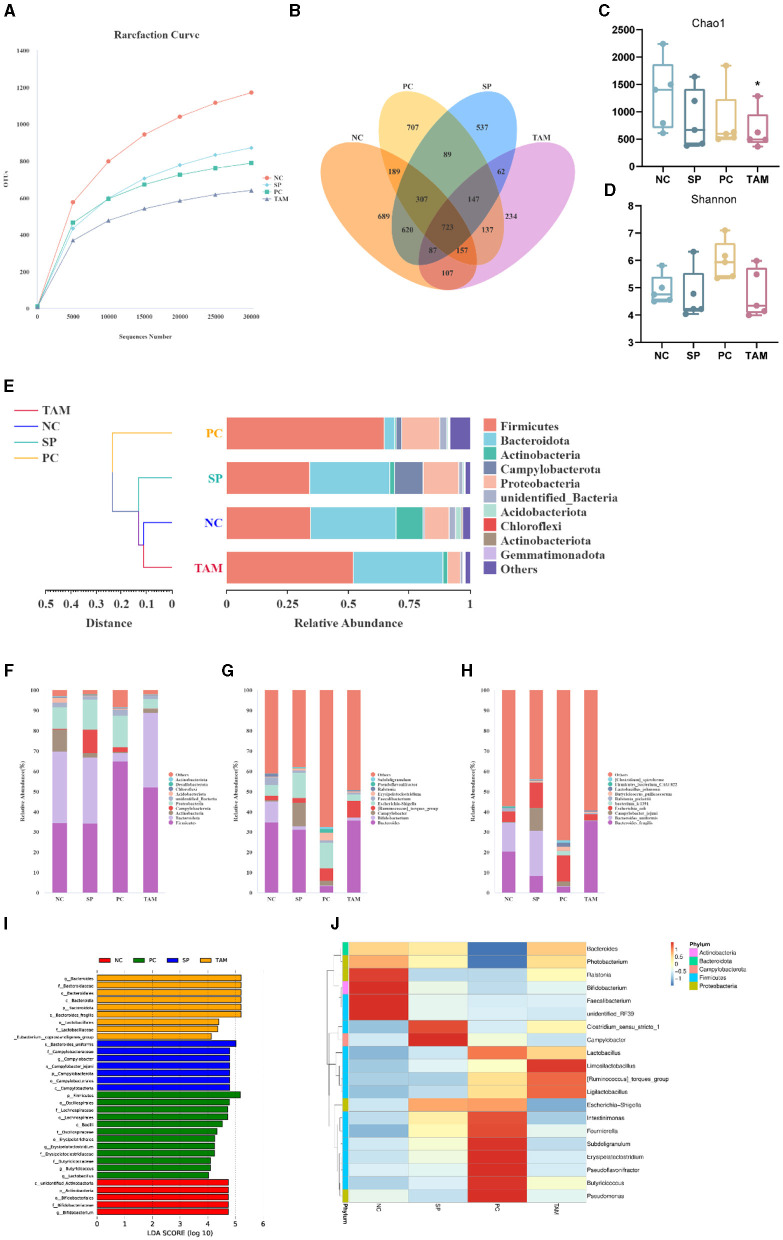
Microbiota of broiler cecum on the 5th day of infection. **(A)** Rarefaction curves of OTUs. **(B)** Venn diagram. **(C, D)** Alpha diversity comparison (Chao1 index and Shannon index). **(E)** UPGMA clustering tree based on weighted Unifrac distances at the phylum level. Relative abundance of species in the top 10 of the intestinal flora at the phylum **(F)**, genus **(G)** and species level **(H)**. **(I)** The histogram of the distribution of LDA values (LDA scores >4). **(J)** Heatmap depicting the relative abundance of 35 dominant microbiota genera at the genus level. All data are expressed as the mean ± SD. The results are presented as the mean ± SD. “*” stands for the comparison with NC; *P < 0.05 by one-way ANOVA.

The aim of this study was to investigate the impact of *S. pullorum* infection and TA treatment on the cecal microbiota of chicks. To achieve this, we sequenced the 16S rRNA gene in samples from four groups: NC, SP, PC, and TAM. Figure 8A shows the rarefaction curves of the samples, indicating adequate sequencing depth and variations in species richness between the groups. The results of the Venn diagram show that there were 689, 537, 707, and 234 unique Operational Taxonomic Units (OTUs) in each respective group. The number of observed OTUs in each group were 2,879, 2,572, 2,456, and 1,654, respectively (Figure 8B). The microbiota's richness and diversity were measured using the Chao1 and Shannon indices. After infection with *S. pullorum*, all groups showed a decrease in Chao1 indices compared to the NC. But only the TAM treatment group showed a statistically significant difference (*P* < 0.05; Figure 8C). The Shannon index did not exhibit any significant differences among the groups (Figure 8D). The UPGMA clustering analysis revealed that PC formed a distinct cluster, whereas NC, SP, and TAM were grouped together on a single branch. It is worth noting that NC and TAM exhibited a closer relationship, as depicted in Figure 8E. The cecal microbiota of chicks from four groups (NC, SP, PC, and TAM) were analyzed at the phylum, genus, and species levels. The main phyla observed in all groups were Firmicutes, Bacteroidetes, Actinobacteria, and Proteobacteria (Figure 8F). The Actinobacteria, though, were only abundant in NC (10.99%) and decreased significantly in SP (2.09%, *P* < 0.05), PC (0.54%, *P* < 0.05), and TAM (1.87%, *P* < 0.05). Additionally, SP had a significantly higher abundance of Campylobacter (11.57%, *P* < 0.05) than NC (0.46%). The genus Bacteroides was dominant in all groups (Figure 8G). Bifidobacterium was significantly lower in SP (1.84%, *P* < 0.05), PC (0.19%, *P* < 0.05), and TAM (1.40%, *P* < 0.05) compared to NC (10.40%). In contrast, Campylobacter was significantly higher in SP (11.56%, *P* < 0.05) than in NC (0.46%). Escherichia-Shigella was more abundant in SP (12.50%) and PC (12.85%) than in TAM (3.00%, *P* < 0.05). The abundance of Ruminococcus torques group was higher in the PC (6.06%) and TAM (8.23%) compared to the NC (2.28%), but the difference was not statistically significant. At the species level (Figure 8H), Bacteroides fragilis (20.43%) and Bacteroides uniformis (14.17%) were the most abundant species in NC, while Bacteroides uniformis (22.14%) prevailed in SP, and Bacteroides fragilis (35.43%) prevailed in TAM. At the genus level, there was a significant increase in *Campylobacter_jejuni* (11.56%, *P* < 0.05) in SP compared to NC (0.49%). Conversely, TAM had significantly lower levels of both *Campylobacter_jejuni* (0.21%, *P* < 0.05) and *Escherichia coli* (3.0%, *P* < 0.05) compared to NC. Linear discriminant analysis (LDA) was used to identify cecal microbial taxa that displays significant differences among the four groups: NC, SP, PC, and TAM. Figure 8I shows the taxa with LDA scores >4, indicating significant differences. All groups exhibited a decrease in Bifidobacterium abundance at the genus level following *S. pullorum* infection compared to NC. Additionally, the SP group showed a higher abundance of Campylobacter. The abundance of *Erysipelatoclostridium, Butyricicoccus*, and *Lactobacillus* was higher in PC, while Bacteroides was more abundant in the TAM group. To visualize the differences in taxa abundance, a heatmap was generated using the top 20 genera by abundance. The SP group had 11 genera that differed from the NC group, the PC group had 18, and the TAM group had 8 (Figure 8J).

## 4 Discussion

The gut mucosa serves as the primary defensive barrier against pathogenic microorganisms (22, 23), playing a crucial role in preventing bacterial infections. Infections caused by *S. pullorum* to significantly disturb the structure and function of gut morphology, leading to considerable mortality rates in poultry (24–26). This issue has caused significant economic losses in the poultry farming industry in developing countries. The study aims to evaluate the protective effects of TA on the integrity and functionality of the intestinal barrier after *S. pullorum* infection in broiler chickens, given the well-documented antimicrobial properties of TA and their potential to modulate the composition of the intestinal microbiota. The immune response of broiler chickens relies on the thymus, spleen, and bursa (27, 28) and the relative weights of these organs can indicate immune function in chickens (29). The study found that infection with *S. pullorum* resulted in a decrease in the weight of the thymus and bursa. In contrast, treatment with TA, preserved the normal development of these organs and also resulted in an increase in spleen weight. These findings are consistent with the results reported by Chen et al. (26), suggesting that TA can enhance the immune function and disease resistance of chicks. Previous studies have also reported a protective effect of TA on the liver (30). The study found that the administration of TA significantly reduced the colonization of *S. pullorum* and mitigated liver damage (31).

The integrity and height of the intestinal villus reflect gut integrity and are fundamental to the gut's barrier function (5, 32). Previous studies have reported that the invasion of *S. pullorum* into chick intestines disrupts the intestinal barrier function (26, 33). Our study confirms these findings, as the intestinal villi of chicks in the SP group were fragmented and shortened. By contrast, the three TA-supplemented groups showed a marked improvement in the morphological structure of the intestine, with the TAM group showing the most improvement. Serum concentrations of lipopolysaccharide (LPS) and diamine oxidase (DAO) are important indicators of intestinal permeability. As previously found, TA significantly reduced serum LPS and DAO levels, indicating its potential to reduce intestinal permeability in chicks. These results suggest that TA helps to preserve the structural integrity of the intestines and mitigate damage caused by *S. pullorum*. The intestinal epithelium is composed of different cell types with different functions: goblet cells, for example, secrete mucus and form a barrier to pathogenic microorganisms; these cells are connected by tight junctions (34–36). The invasion of *S. pullorum* disrupts the mucus layer and facilitates the spread of harmful substances (37–39). The protective effect of TA on the intestinal barrier function of broiler chickens infected with *S. pullorum* was evaluated in this study based on the antimicrobial properties of TA and the regulation of tight junction proteins (13, 20). The gene expression of intestinal claudin-1, occludin, ZO-1, and MUC2, as well as the number of goblet cells, were found to be significantly reduced as a result of *S. pullorum* infection. TA supplementation improved the goblet cell count and increased the expression of tight junction proteins and MUC2, with the most significant effect observed in the TAM group. *S. pullorum* is a facultative intracellular parasitic bacterium that primarily induces Th2-type immune responses, which may contribute to its persistence and infection (40–42). Secretory immunoglobulin A (sIgA) is a crucial marker of intestinal immunity (43, 44). Supplementation with TA enhanced the mRNA expression of Th1 and Th2 cytokines and increased jejunal sIgA secretion. These findings suggest that TA improves the intestinal mucosal barrier function and antimicrobial capacity. The composition of intestinal flora is closely linked to the function of the intestinal barrier and plays a crucial role in the host's defense against pathogenic microorganisms and energy provision (45, 46). Previous studies have indicated that *S. pullorum* invasion disrupts gut flora, although the exact effects remain to be elucidated (33, 47, 48). Our study confirms these findings, demonstrating a significant decrease in gut microbial diversity in chicks infected with *S. pullorum*. Notably, the addition of TA supplementation did not significantly increase microbial diversity, but it did align more closely with the NC group in terms of evolutionary levels. This may be due to the selective bacteriostatic effect of TA. To further analyze the dominant flora at different levels, changes at the phylum level were investigated. The study found a significant increase in the relative abundance of Proteobacteria in the SP group of chicks, which is often associated with many diseases (49, 50). Furthermore, the pathogen Campylobacter, a which is known to harm the gut mucosa and increase gut permeability, was identified as particularly harmful to humans (51, 52). The study conducted on SP group broilers revealed a significant increase in the relative abundance of both Campylobacter and Escherichia–Shigella, which is consistent with the findings of Huang et al. (47). These results suggest that *S. pullorum* disrupts the gut flora and promotes the growth of harmful bacteria. Supplementation with TA, resulted in a significant decrease in the relative abundance of Campylobacter and a significant increase in Bacteroides fragilis. Bacteroides fragilis has potential as a probiotic, as it enhances macrophage phagocytosis and promotes a Th1-type immune response (53). This may further inhibit *S. pullorum* enterica colonization in chickens with dysentery. The results of the heat map indicate that Lactobacillus (54), Ligilactobacillus (55), and Limosilactobacillus (56), which are closely related to gut homeostasis, were enriched in the TAM group. This suggests that TA can effectively prevent the colonization of harmful bacteria in the intestine, promote the growth of beneficial microorganisms, enhance immune function, and maintain the structural and functional integrity of the intestine.

## 5 Conclusion

In conclusion, our study provides evidence that TA supplementation can effectively mitigate *S. pullorum* colonization, reduce chick mortality and improve body weight. These effects are mainly mediated by upregulation of genes associated with gut barrier function and modulation of the gut microbiota. Our findings provide a novel approach for the clinical management of PD.

## Data availability statement

The original contributions presented in the study are publicly available. This data can be found at the National Center for Biotechnology Information (NCBI) using accession number PRJNA1108228.

## Ethics statement

The animal study was approved by the Ethics Committee of Sichuan Agricultural University. The study was conducted in accordance with the local legislation and institutional requirements.

## Author contributions

JZ: Conceptualization, Data curation, Formal analysis, Investigation, Methodology, Resources, Validation, Visualization, Writing – original draft, Writing – review & editing. HL: Conceptualization, Data curation, Formal analysis, Investigation, Methodology, Resources, Validation, Visualization, Writing – original draft, Writing – review & editing. PX: Conceptualization, Data curation, Formal analysis, Investigation, Methodology, Resources, Validation, Visualization, Writing – original draft, Writing – review & editing. JX: Formal analysis, Investigation, Methodology, Visualization, Writing – original draft, Writing – review & editing. JF: Data curation, Formal analysis, Validation, Visualization, Writing – original draft, Writing – review & editing. XZhon: Data curation, Formal analysis, Validation, Visualization, Writing – original draft, Writing – review & editing. XZhou: Validation, Visualization, Writing – original draft, Writing – review & editing. XS: Formal analysis, Investigation, Supervision, Writing – original draft, Writing – review & editing. XZha: Validation, Visualization, Writing – original draft, Writing – review & editing. YZ: Validation, Visualization, Writing – original draft, Writing – review & editing. LL: Validation, Visualization, Writing – original draft, Writing – review & editing. RJ: Validation, Visualization, Writing – original draft, Writing – review & editing. YF: Validation, Visualization, Writing – original draft, Writing – review & editing. ZL: Validation, Visualization, Writing – original draft, Writing – review & editing. ZY: Conceptualization, Formal analysis, Funding acquisition, Investigation, Methodology, Project administration, Resources, Supervision, Writing – original draft, Writing – review & editing.
